# Supplementary material for: The Effect of the PhoP/PhoQ System on the Regulation of Multi-Stress Adaptation Induced by Acid Stress in Salmonella Typhimurium
Source: Foods. 2024 May 15;13(10):1533. doi: 10.3390/foods13101533 (PMC11121531; doi:10.3390/foods13101533)
Supplement: Supplementary file 1 [file foods-13-01533-s001.zip › foods-2988377-supplementary.pdf]

Supplementary Materials:

Table S1. Summary of RNA-seq date

Sample	Raw.reads	Clean.reads	Raw.bases	Clean.bases	Error.rate	Q20	Q30	GC.content
WT1	7680778	7621796	1.2G	1.1G	0.03	97.87	94.06	52.76
WT2	6833978	6779136	1.0G	1.0G	0.03	97.81	93.9	52.58
WT3	6623380	6533038	1.0G	1.0G	0.02	97.97	94.28	52.81
phoP1	6684334	6638716	1.0G	1.0G	0.03	97.75	93.78	52.71
phoP2	7179382	7124722	1.1G	1.1G	0.03	97.94	94.22	52.78
phoP3	7647970	7596298	1.1G	1.1G	0.03	97.9	94.14	52.83
